# Network meta-analysis: application and practice using Stata

**DOI:** 10.4178/epih.e2017047

**Published:** 2017-10-27

**Authors:** Sungryul Shim, Byung-Ho Yoon, In-Soo Shin, Jong-Myon Bae

**Affiliations:** 1 Institute for Clinical Molecular Biology Research, Soonchunhyang University Hospital, Seoul, Korea; 2 Department of Orthopaedic Surgery, Seoul Paik Hospital, ,Inje University College of Medicine, Seoul, Korea; 3 Department of Education, Jeonju University, Jeonju, Korea; 4 Department of Preventive Medicine, Jeju National University School of Medicine, Jeju, Korea

**Keywords:** Network meta-analysis, Treatment outcome, Mixed treatment comparison, Biostatistic

## Abstract

This review aimed to arrange the concepts of a network meta-analysis (NMA) and to demonstrate the analytical process of NMA using Stata software under frequentist framework. The NMA tries to synthesize evidences for a decision making by evaluating the comparative effectiveness of more than two alternative interventions for the same condition. Before conducting a NMA, 3 major assumptions—similarity, transitivity, and consistency—should be checked. The statistical analysis consists of 5 steps. The first step is to draw a network geometry to provide an overview of the network relationship. The second step checks the assumption of consistency. The third step is to make the network forest plot or interval plot in order to illustrate the summary size of comparative effectiveness among various interventions. The fourth step calculates cumulative rankings for identifying superiority among interventions. The last step evaluates publication bias or effect modifiers for a valid inference from results. The synthesized evidences through five steps would be very useful to evidence-based decision-making in healthcare. Thus, NMA should be activated in order to guarantee the quality of healthcare system.

## INTRODUCTION

As newly developed drugs conducted the third stage of randomized clinical trials (RCT) are approved for marketing, and enter into the armamentarium of treatment, there is a need for comparative effectiveness research (CER) to evaluate the effectiveness of drugs used for the same treatment goal and meta-analysis to synthesize the results of the CER [1]. Conventional meta-analysis on the treatment effects of new drugs is conducted on the effect size based on pairwise head-to-head direct comparison, but data from direct comparisons are relatively limited [2]. In contrast, the necessity for indirect comparisons among various drugs of the same efficacy used in clinical practice became greater [3,4]. Accordingly, an analytic approach called network meta-analysis (NMA) was developed to include in the meta-analysis not only direct comparisons, but also indirect comparisons based on logical inference; in the latter case, no comparisons are actually performed [3,5-10].

Statistical approaches to NMA are largely classified as frequentist and Bayesian frameworks [7]. Because part of NMA has indirect, multiple comparisons, Bayesian framework seems logically more valid, and 60-70% of NMA studies have taken a Bayesian approach [6,11-13]. However, if the prior probability is not established in the study hypothesis, Bayesian analysis poses many limitations for ordinary researchers using NMA because the problem of establishing prior probability is rather more complex than the problem of testing the research hypothesis, that is, the original purpose of the analysis [7]. In 1997, Bucher et al. [14] proposed an NMA approach based on the frequentist framework using random-effects models. Since then, many methodological developments have taken place [7,15], and articles introducing SAS (https://www.sas.com/en_us/home.html) and Stata (http://www.stata.com) program commands have been presented [12,15,16].

From 2008 onwards, the number of publications based on NMA increased at a rapid pace [2-4,15]. In 2011, the International Society for Pharmacoeconomics and Outcomes Research (ISPOR) defined the concepts related to NMA and established guidelines relating to methodological and statistical issues to help researchers conduct NMA in a valid manner [7,17]. Furthermore, ‘PRISMA (Preferred Reporting Items for Systematic Reviews and Meta-analyses) Network Meta-Analysis checklist’, as a guideline for reporting NMA research outcomes, was also developed [7,18].

As NMA has features of multivariate multi-level analysis fundamentally, clinical researchers, as well as epidemiologists and statisticians, should understand them sufficiently well to perform NMA [19]. Hence, in this article, the terms used in NMA are defined, relevant statistical concepts are summarized, and the NMA analytic process based on the frequentist framework is illustrated using Stata program and an actual example.

## INTRODUCTION OF RELATED CONCEPTS

### Definition of terms

Since Bucher et al. [14] proposed the concept of indirect treatment comparison (ITC) on treatment effect in 1997, such terms as ‘multiple treatment comparison’ meaning comparison of several treatments [20] and ‘mixed treatment comparison’ (MTC) meaning a combination of direct and indirect comparisons [6] have been introduced.

**Figure 1.**
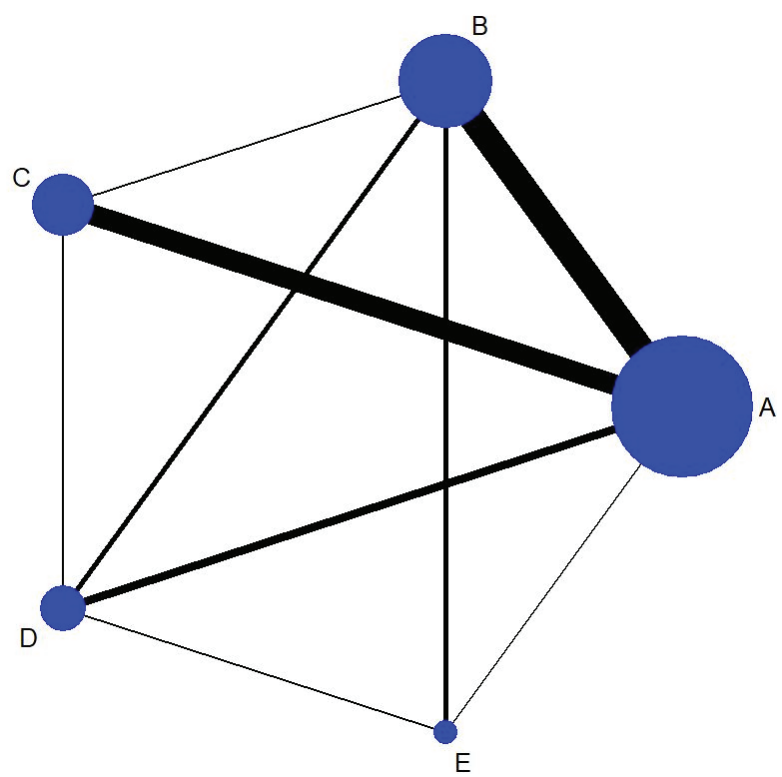
Network geometry. When the obejctive is to examine comparative effectiveness of B-C among the treatments A, B, and C, a closed loop is present if research data comparing all 3 pairs (A-B, B-C, and A-C) exist.

However, at present, NMA is primarily used to mean a research effort to synthesize the results obtained by comparing several studies which examined multiple treatments [3,5,7,16,21]. ISPOR [7] defines NMA as a comparison of the effectiveness of 2 or more treatments, and categorizes the comparison type as MTC if the network geometry shows a closed loop and ITC if it does not (Figure 1).

### Relevant assumptions

Meta-analysis is a statistical methodology to synthesize the results of several studies, and overall effect size is valid only if various a priori assumptions are satisfied [22]. Furthermore, NMA requires more strict methodological, logical, and statistical assumptions [23,24], about similarity, transitivity, and consistency, respectively [5,12,17,19,25,26]. In NMA, whether each of these is satisfied must also be examined [8,10,19].

#### Similarity

To compare among the clinical trial studies extracted for analysis, similarity in the methodology used in the studies must be assumed [5,27]. Similarity is qualitatively assessed on each of the selected articles from a methodological point of view, and is not a hypothesis to be tested statistically. To examine similarity, the population, intervention, comparison, and outcome (PICO) technique is used [26]. Specifically, similarity among the studies extracted for analysis is examined for the following 4 items: clinical characteristics of study subjects, treatment interventions, comparison treatments, and outcome measures. If the similarity assumption is not satisfied, not only are the other two assumptions negatively affected [9], but there is also a need to check for the heterogeneity error [10,23].

#### Transitivity

Transitivity covers the validity of the logical inference, while similarity relates to the methodological feasibility of comparing. To explain simplistically, if direct comparisons of 3 drugs—A, B, and C—treating the same illness found that A was more effective than B, and B was more effective than C, then A can be expected to be more effective than C, even though the two were never directly compared. Such transitivity should be satisfied for all cases in an NMA [4,5,28].

If the researcher compares the outcomes of direct and indirect comparisons according to logical inference, the satisfaction of the transitivity assumption can be examined objectively. Statistical assessment of the outcomes of direct and indirect comparisons is called consistency [4,5,10]. If inconsistency is observed, non-transitivity should be suspected [29]. If non-transitivity is suspected, the presence of effect modifiers influencing treatment effect should first be examined [9,27,30].

#### Consistency

Consistency, an objective measure of transitivity, means that the comparative effect sizes obtained through direct and indirect comparisons are consistent. Hence, consistency is statistically examined transitivity [5,7,24,31], and some researchers call it coherence [8,21,32]. For this reason, the assumptions of transitivity and consistency can be regarded as similar [9,33,34]. However, the authors of this article differentiate transitivity from consistency to emphasize that the perspectives used are logical and statistical, respectively.

According to a summary of techniques to check the consistency assumption utilized in the existing NMA studies [2], the techniques are categorized as those evaluating the fitness of statistical models [34,35] and those assessing the logical structure of graphs [29]. The model [Y] presented in the Stata program simultaneously considers the level of heterogeneity [H] (which should be examined in any meta-analysis) and the level of inconsistency [C] (for conducting multiple comparisons within a network), as well as the size of the treatment effect [D] of interest [16]. The equation that considers within-study variance (E), Y= D+H+C+E, is called the inconsistency model. If the level of inconsistency is zero, that is, [C= 0], it is considered a consistency model.

Consistency is statistically evaluated based on the confidence interval of the difference in comparative effect size between direct and indirect comparisons [10,32], and inconsistency is observed in approximately 1/8 of NMA studies [23]. It is very important to identify the cause of inconsistency [1,10,23]. Ioannidis [36] proposed 4 kinds of causes of inconsistency—chance, bias in headto-head comparison, bias in indirect comparison, and genuine diversity—and Higgins et al. [35] classified the causes of inconsistency into loop inconsistency, which refers to a difference between direct and indirect comparisons, and design inconsistency, which refers to a difference due to different sets of treatments being compared.

Stata tests for inconsistency have 2 levels [12,16]. The first is a global approach to test for overall inconsistency, in which the level of inconsistency is computed according to the type of between-treatment comparison for all cases and then the values are used to test for global linearity via the Wald test. The second is a local approach, in which each treatment is individually examined (nodesplitting) and the outcomes of direct and indirect comparisons are statistically tested.

Researchers should pay attention to the consistency assumption and explore the presence of effect modifiers causing overall inconsistency in the global approach [12,37,38]. To examine them, sensitivity analysis is utilized [3,30], and if it is determined that an effect modifier is present, performing meta-regression is recommended to adjust the corresponding variable [3,5,38].

### Network geometry

Network geometry is a diagram showing the interactions among the articles included in NMA [39]. The diagram provides important information in establishing analytic strategies and interpreting the results [5,8,39], and so it is strongly recommended to use network geometry in presenting the NMA analysis results [1,18]. One of NMA’s features is that network geometry may change with an addition of new research outcomes or new treatments in the comparison set [39].

## ILLUSTRATION OF STATA APPLICATION

### Preparing for analysis: information extraction and network meta-analysis support program installation

The following research question was formulated to illustrate how to perform NMA: whether transfusion rate in total hip joint replacement is different depending on the method of tranexamic acid administration. During the literature search process, 25 articles were selected and the extraction results are listed in Appendix 1. Drug administration was classified into the following 5 treatments: placebo (A); IV_single use (B); IV_double use (C); topical use (D); and a combination of IV and topical use (E).

As shown in Appendix 1, a long form to code the sample size for each treatment group in a study is recommended because this format makes it easy to understand the commands and also makes it easy to edit data, if necessary.

**Figure 2.**
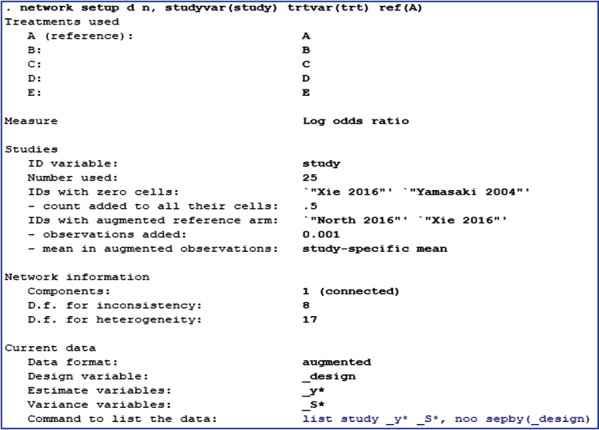
Results of network setup order.

To perform NMA using Stata, the network package should first be installed [16]. Then, the variables in the analysis should be specified by typing the command < network setup d n, studyvar (study) trtvar(trt) ref(A)> . In the command, < network setup> means that network package is used for analysis. The number of events (< d> ) and the total sample size (< n> ) are entered, in this order. After a comma, the relevant options are entered; < studyvar> refers to the variable for study title; < trtvar> is the variable for treatments; and < ref> is the variable for the reference treatment among the treatments. The command corresponding to the data organized in Appendix 1 is found in Figure 2.

According to Figure 2, the reference treatment is A (placebo). Of the 25 studies, Xie 2016 and Yamasaki 2004 included cells with d= 0, and Stata replaced them with a default value of 0.5. Consequently, 0.5 was assigned to both the intervention and control groups, which increased the sample size per treatment by 1. Also, for studies with no information on the reference treatment, A, as in North 2016 and Xie 2016, Stata generates a tiny amount of data in the reference arm as a default. This practice is called augmented method, and is advantageous because the overall effect size is not affected, errors in the equations can be reduced, and all extracted studies are utilized in the analysis.

### Step 1: generating network geometry

The command to draw a network geometry to explore comparative relationships among treatments is < network map > , and Figure 1 shows the outcome of the current example. The size of the 5 nodes—one for each treatment—indicates the number of studies included in the corresponding nodes, while the thickness of the lines connecting 2 nodes indicates the amount of relevant data. Also, all 5 nodes were closed, which confirms that MTC analysis can be performed. To examine the contributions of individual treatments in a table form, the command < netweight> is used.

### Step 2: testing for inconsistency

**Figure 3.**
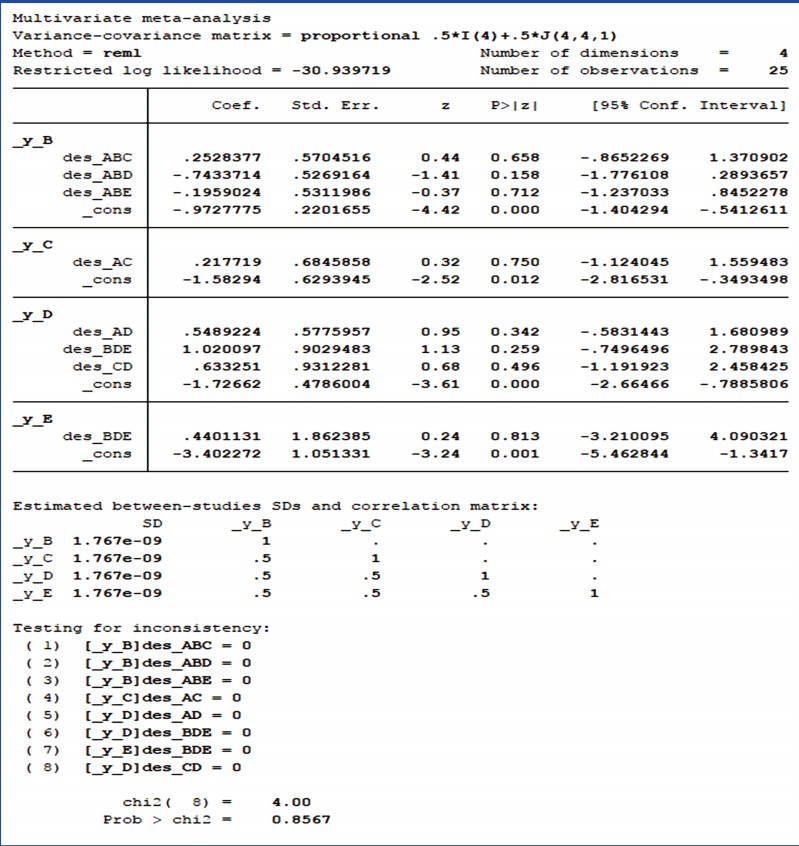
Results of test for inconsistency.

This step in NMA is to statistically test whether the consistency assumption among 3 NMA assumptions is satisfied. To check for overall inconsistency, the command < network meta inconsistency> is used for the inconsistency model provided in Stata, and Figure 3 shows the results of this case study. The p-value displayed at the bottom of Figure 3 is the result of testing for inconsistency at the overall level. Based on the p-value, the null hypothesis cannot be rejected and the consistency assumption could be accepted at the overall level of each treatment.

Next, the command < network sidesplit all> is used for the local test on loop inconsistency. Table 1 shows the results of the local inconsistency test in this case study, listing the size of differences for each treatment and the statistical test results. None of the treatments showed statistical significance. Because inconsistency was found to be absent in both global and local tests, the consistency assumption was accepted.

### Step 3: creating plots and league table of effect size by treatment

**Figure 4.**
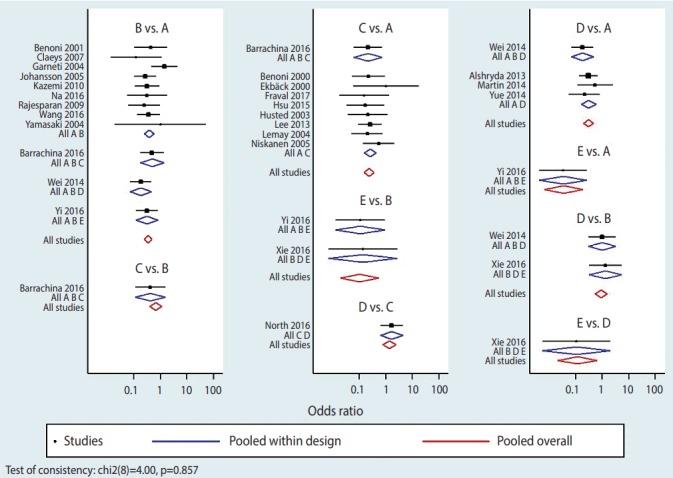
Network forest plot. A, placebo; B, IV_ single; C, IV_double; D, topical; E, combination.

To display effect sizes in a plot and a league table, the outcomes should first be stored in memory using the command < network meta consistency> . There are 2 ways in NMA to graphically represent effect size by study and by treatment: network forest plot (NFP) and interval plot. To generate NFP in Stata, the command < network forest, msize (*0.15) diamond eform xlabel (0.1 1 10 100) colors (black blue red) list> is typed. The main command to generate forest plots is < network forest> , and options are specified after a comma. Among the various options, < diamond> uses a diamond shape to show summary effect sizes and < eform> generates transformed indices to make it easy to interpret the forest plot. Other options are there to help in easily visualizing the graph: < msize (*0.15)> decreases the value of individual studies’ effect size by 0.15 times; < xlabel (0.1 1 10 100)> sets the unit on the x axis; and < colors (black blue red)> sets the colors of the effect of each study within a treatment in the comparison set, the pooled effect of a treatment in the comparison set (also called “pooled within design”), and the pooled overall effect (also called “pooled overall”) as black, blue, and red, respectively (Figure 4).

A wide range of information is obtained in an NFP. First, it provides information on the effect size of each study and each treatment. The pooled effect of each treatment in the comparison set (blue color) shows the results of the test for the inconsistency model, And the pooled overall effect (red color) shows the result of the test for the consistency model. Second, the p-value displayed in the lower left of the plot is congruent with the result of the global test on inconsistency, which confirms that the consistency is accepted. Third, heterogeneity among individual studies within a treatment can be visually inspected. Moreover, based on the similarity between the size of pooled effect of each treatment in the comparison set (blue color) and the size of pooled overall effect (red color), it can be determined whether the consistency model is supported.

**Figure 5.**
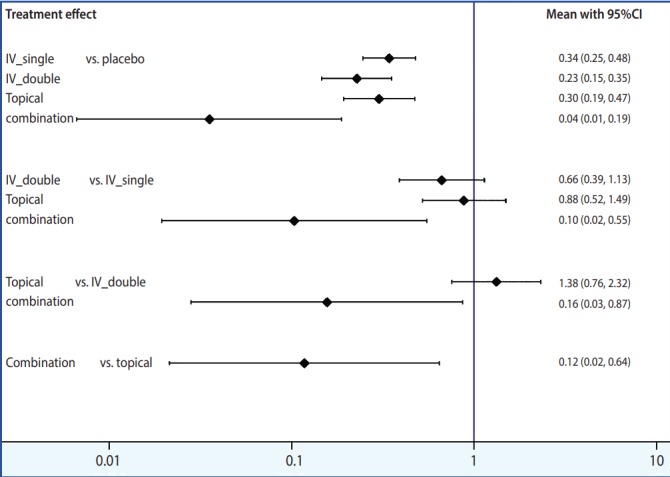
Interval plot. CI, confidence interval.

Although useful information is provided by NFP, readability suffers if the number of articles included in the analysis or treatments in the comparison set is large. In such a case, it is advised to generate interval plots, by typing the command < intervalplot, eform null (1) labels (Placebo IV_single IV_double Topical Combination) separate margin (10 8 5 10) textsize (2) xlabel (0.01 0.1 1 10)> . The main command is < intervalplot> ; < eform> transforms the original logarithmic data into indices for easier interpretation; < null (1)> inputs 1, the value indicating statistically significant difference in a ratio like odds ratio; <label> defines how treatments should be labeled; < separate> and < margin> set the ranges to generate easy-to-read plots, the values of which should be appropriately determined by the user because they vary greatly depending on the number of articles as well as the number of treatments in the comparison set. Figure 5 shows the interval plot obtained by typing the commands discussed above. It is relatively intuitive to compare the effect sizes of individual treatments and very easy to interpret the results. A network league table, shown in Appendix 2, can be created based on the outcome comparing the effect sizes of treatments, which is produced in the Stata result window after the command < intervalplot> is typed.

### Step 4: determining relative rankings of treatments

Once comparative effectiveness of the treatments has been evaluated through the previous steps, the next step is to rank order the treatments to identify superiority [12]. In other words, the treatment interventions showing the most superior treatment effect are evaluated.

Stata supports two commands—network rank and surface under the cumulative ranking (SCURA)—to rank order treatments. There is little difference in the outcome between the commands, but it is easier to use network rank, for which the command < network rank min, line cumulative xlabel (1/4) seed (10000) reps (10000) meanrank> is typed. The main command is < network rank> , and < min (or max)> specifies whether superiority should be determined by using ascending or descending order of effect size. In this case study < min> was used because a treatment is more effective as the effect size compared to the reference treatment (placebo, A) is smaller.

**Figure 6.**
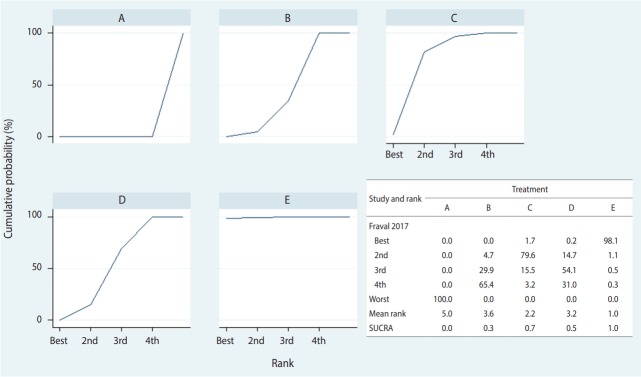
Results of network rank test. A, placebo; B, IV_single; C, IV_double; D, topical; E, combination; SCURA, surface under the cumulative ranking.

As shown in Figure 6, the probability of treatment E (combination) being the best is approximately 98.1%, and the probability for it to be at least the second best is 99.2%. In the SCURA, the surface area for treatment E reaches almost 100%, confirming again that it is the best intervention [40]. The SUCRA command is used for more precise estimation of cumulative ranking probabilities. Based on the SUCRA results, treatment E (combination) is followed by C (IV_double), D (topical), B (IV_single), and A (placebo). A clinical interpretation of the results is that the administration of tranexamic acid, in combination with IV and topical use is recommended to ensure maximum decrease in the probability of transfusion in total hip joint replacement.

### Step 5: checking for publication bias

To check for publication bias in NMA, a network funnel plot is created. Because the Stata network package does not directly support the generation of network funnel plots, the data should first be transformed, as demonstrated in Appendix 2. To do so, data should be generated by typing the network forest command with the list option, and the comparative effect size (diff) and standard error (se) are summarized for each pair of treatments within individual articles that are directly compared with each other (t1, t2).

After the data shown in Appendix 3 are uploaded in a new Stata window, the command < netfunnel diff se t1 t2, random bycomparison > is typed to generate network funnel plots. Here, the main command is < netfunnel> ; < diff> is comparative effect size between treatments in logarithmic scale and < se> indicates se; < random> in the option list means the use of a random effect model, and < bycomparison> is to color code treatments. If the user wants to generate selective funnel plots with respect to placebo A, the command < netfunnel diff se t1 t2 if t1= = “A”, random bycomparison> is typed. Once the plots are generated, publication bias is visually inspected using the criterion of symmetry. It is advised to consider performing sensitivity analysis, if necessary.

## DISCUSSION AND SUGGESTIONS

So far, the application of NMA within frequentist framework has been demonstrated. Below, we list points for researchers to consider when performing NMA and suggestions for those points.

First, NMA is for direct and indirect comparisons in a set of treatments and it is limited to the results of RCT studies [5]. At the current advancement of the analytic technique, it is not recommended to apply NMA to the results of observational research like cohort study and case-control study. Also, the fact that researchers, funding agencies, the types of drugs approved for marketing in different countries, and the research ethics committees are operated, etc., could affect the RCT research issues and outcomes, should be factored in [41].

Second, the 3 assumptions to be satisfied in NMA should be examined meticulously [25,42]. Of the assumptions, practical decisions from a clinician point of view should be made on the similarity and transitivity assumptions [42]. If inconsistency is concluded via statistical testing, effect modifiers should be identified by utilizing network geometry to thoroughly investigate the relationships among the selected articles [37]. Then, NMA meta-regression should be performed and the results before and after the adjustment with the effect modifiers should be evaluated in order to derive clinically valid conclusions [5,31].

Third, the presence of bias due to small-scale studies should be considered [8]. Studies with a small number of subjects can not only cause publication bias [43], but also generate a relatively large treatment effect [44]. Therefore, it is recommended to evaluate publication bias by using a random effect model [7] and performing sensitivity analysis [3,30].

Fourth, the application of Bayesian framework may be considered, as limitations exist in NMA based on frequentist framework [6,7]. If the ultimate goal of an NMA study is to make a medical decision and predict the outcome under uncertainty, Bayesian framework is more appropriate [11-13]. At present, several statistical programs supporting Bayesian analysis are available, including WinBUGS (https://www.mrc-bsu.cam.ac.uk/software/bugs/) [12].

Evidence synthesized through a systematic review based on meta-analysis is the most powerful with respect to scientific persuasion [45]. Moreover, the outcomes of NMA—an analysis that synthesizes comparative effectiveness through direct and indirect comparisons among treatment interventions with the same treatment goal and rank orders them—have great significance in evidence-based decision making in health care [3,5,46]. Thus, the use of NMA should be facilitated to enhance the quality of health care in Korea, and it is expected that this article will motivate Korean researchers to facilitate the application and practice of NMA.

## Figures and Tables

**Table 1.** Inconsistency test between direct and indirect treatment comparisons in mixed treatment comparison

Side	Direct		Indirect		Difference		p>z
	Coefficient	SE	Coefficient	SE	Coefficient	SE	
A B	-1.083	0.174	-0.877	0.620	-0.206	0.636	0.746
A C	-1.388	0.247	-1.869	0.493	0.481	0.542	0.375
A D	-1.378	0.265	-0.738	0.413	-0.640	0.479	0.182
A E	-3.425	0.940	-3.221	1.005	-0.204	0.937	0.828
B C	-0.894	0.655	-0.312	0.297	-0.581	0.715	0.416
B D	0.099	0.462	-0.241	0.329	0.340	0.567	0.548
B E	-2.152	0.881	-2.615	1.087	0.463	0.896	0.605
C D	0.490	0.492	0.177	0.350	0.313	0.604	0.605
D E	-2.550	1.254	-1.956	0.958	-0.595	1.314	0.651

SE, standard error; A, placebo; B, IV_single; C, IV_double; D, topical; E, combination.

## Appendix 1.

The extracted information from the selected articles for conducting a network meta-analysis using Stata

## Appendix 2.

A network league table based on the network meta-analysis from data in Appendix 1

## Appendix 3.

The raw data for drawing a network funnel plot from data in Appendix 1
