# Supplementary material for: Network meta-analysis: application and practice using Stata
Source: Epidemiol Health. 2017 Oct 27;39:e2017047. doi: 10.4178/epih.e2017047 (PMC5733388; doi:10.4178/epih.e2017047)
Supplement: Supplementary file 1 [file epih-39-e2017047-supplementary.pdf]

Volume: 39, Article ID: e2017047
https://doi.org/10.4178/epih.e2017047

<방법론>

네크워크 메타분석: STATA를 활용한 적용과 실제

Network Meta-analysis: Application and Practice Using STATA

심 성 률[1], 윤 병 호[2], 신 인 수[3], 배 종 면[4]

[1]순천향대학교 서울병원 임상분자생물학연구소, [2]인제대학교 서울백병원 정형외과,
[3]전주대학교 사범대학 교육학과, [4]제주대학교 의학전문대학원 예방의학교실

교신저자: 배종면
jmbae@jejunu.ac.kr
Tel: 064-755-5567
제주도 제주시 대학로 66

Running Title: Network meta-analysis

Volume: 39, Article ID: e2017047
https://doi.org/10.4178/epih.e2017047

Abstract

This review aimed to arrange the concepts of a network meta-analysis (NMA) and to demonstrate the analytical process of NMA using STATA software under frequentist framework. The NMA tries to synthesize evidences for a decision making by evaluating the comparative effectiveness of more than two alternative interventions for the same condition. Before conducting a NMA, 3 major assumptions—similarity, transitivity, and consistency—should be checked. The statistical analysis consists of 5 steps. The first step is to draw a network geometry to provide an overview of the network relationship. The second step checks the assumption of consistency. The third step is to make the network forest plot or interval plot in order to illustrate the summary size of comparative effectiveness among various interventions. The fourth step calculates cumulative rankings for identifying superiority among interventions. The last step evaluates publication bias or effect modifiers for a valid inference from results. The synthesized evidences through five steps would be very useful to evidence-based decision-making in healthcare. Thus, NMA should be activated in order to guarantee the quality of health care system.

Keywords: Network Meta-analysis, Multiple treatments meta-analysis, Mixed treatment comparison, Consistency, Transivity

Volume: 39, Article ID: e2017047
https://doi.org/10.4178/epih.e2017047

서론

새로이 개발되는 신약은 3상 무작위 배정 임상시험 (randomized clinical trials, RCT)를 거쳐 시판허가를 받아 진료현장에 도입되면서, 같은 치료목적을 가진 여러 약들 간에 효과를 상호 비교하는 비교효과연구 (comparative effectiveness research)와 비교분석한 결과들을 통합하기 위한 메타분석 (meta-analysis)의 필요성이 제기되었다 [1]. 신약의 치료효과에 있어 전통적인 메타분석은 비교군과 짝지은 직접 비교 (pair-wise head-to-head direct comparison)의 효과크기에 대하여 메타분석을 시행하지만, 직접 비교한 결과의 데이터가 상대적으로 부족하다 [2]. 반면, 동일한 약효로 임상 현장에서는 사용되고 있는 여러 치료약들에 있어 간접적 (indirect)인 비교가 더 요구되게 되었다 [3,4]. 이에 따라 기존의 직접 비교뿐만 아니라, 실제로 수행하지 않았지만 논리추론에 따른 간접적 비교도 메타분석에 포함할 수 있도록 개발된 것이 바로 네트워크 메타분석 (network meta-analysis, 이하 NMA)이다 [3, 5-10].

NMA 에 대한 통계학적 접근은 빈도주의 틀 (Frequentist framework)과 베이지언 틀 (Bayesian framework)로 대분한다 [7]. NMA 분석에 간접적 (indirect)이며

Volume: 39, Article ID: e2017047
https://doi.org/10.4178/epih.e2017047
다중적 (multiple) 비교를 담고 있어서 베이지언 틀이 논리적으로 보다 타당하며, NMA을 적용한 논문의 60~70퍼센트 정도가 베이지언 접근을 사용하고 있다 [6, 11-13]. 그러나 베이지언 분석은 연구가설에서 사전확률이 확립되어 있지 않다면 사전확률을 설정하는 문제가 오히려 본래 분석하고자 하는 연구가설 검정보다 더 복잡하여 일반 연구자들이 접근하기에는 많은 한계를 지닌다 [7]. 한편 1997년 Bucher 등 [14]이 랜덤효과 모델 (random-effects models)을 이용한 빈도주의 틀 (frequentist framework) 분석을 제안한 이후 방법론적 개발이 되면서 [7,15], SAS (https://www.sas.com/en_us/home.html)와 STATA (http://www.stata.com) 프로그램 명령어를 소개하는 논문들이 발표되었다 [12,15,16].

한편 NMA를 적용한 논문들의 발간이 2008년 이후 급증하는 가운데 [2-4, 15], 2011년도 국제약물경제학및성과연구학회 (International Society for Pharmacoeconomics and Outcomes Research, ISPOR)는 NMA 의 타당한 수행을 위해 관련한 개념들을 정의하고, 연구방법론 및 통계학적 이슈들을 정립하였다 [7,17]. 나아가 NMA 연구 결과의 보고 지침 (Preferred Reporting Items for Systematic Reviews and Meta-analyses Network Meta-Analysis checklist)까지 개발되었다 [7,18].

Volume: 39, Article ID: e2017047
https://doi.org/10.4178/epih.e2017047

그런데 NMA는 기본적으로 다변량 다층분석(multivariate multi-level analysis)의 속성을 지니고 있어, 역학자, 통계학자뿐만 아니라 임상연구가들도 충분히 이해해야만 한다 [19]. 이에 따라 본 논고는 네트워크 메타분석의 용어 정의와 통계 관련 개념들을 정리한 다음, 실제 예제를 정하여 빈도주의 틀에 따라 STATA 프로그램을 통한 NMA 분석 과정을 설명하고자 한다.

Volume: 39, Article ID: e2017047
https://doi.org/10.4178/epih.e2017047

관련 개념들 소개

가. 용어 정의

1997년 Bucher 등[14] 은 치료효과에 대한 간접 비교 (indirect treatment comparison, ITC)에 대한 개념을 제시한 이후, 여러 치료들의 비교라는 뜻에서 다중 비교 (multiple treatment comparison) [20], 직접과 간접을 같이 비교한다는 혼합 비교 (mixed treatment comparison, MTC) [6] 등의 용어들이 제시되었다.

그러나 다수의 치료를 가진 여러 연구들을 비교하여 그 결과를 종합한다는 의미에서 현재는 NMA란 용어로 통합되었다 [3,5,7,16,21]. ISPOR [7]은 2개 이상의 치료 효과를 비교하는 경우를 NMA 라 정의하면서, Fig 1. 같은 네트워크 지형도 (network geometry)에서 닫힌 loop를 구성하면 MTC, 그렇지 않으면 ITC라 구분한다.

나. 관련한 가정 (assumptions)

메타분석은 여러 연구 결과들을 종합하는 통계방법으로, 사전에 여러 가정들을 충족하여야만 종합효과크기(overall effect size)의 타당성을 확보할 수 있다 [22]. 더군다나 2개 이상의 치료효과를 비교하는 NMA의 경우는 연구방법론적, 논리추론적, 통계분석학적으로 보다 더 엄격한 가정들이 요구된다 [23,24]. 즉, 연구방법론적 측면의 유사성 (Similarity), 논리추론적 측면의 이행성 (Transitivity), 통계분석적 측면의 일관성 (Consistency)이 NMA 에서 요구되는 주요 가정들이다 [5,12,17,19,25,26]. NMA 분석 과정에서 이들 가정들이 충족되는가를 반드시 점검해야 한다 [8,10,19].

(1) 유사성 (Similarity)

분석 대상이 되는 임상시험 논문들 간에 상호비교가 가능하려면, 연구방법론적으로 유사성이 전제되어야 한다 [5,27]. 따라서 유사성은 연구방법론적인 차원에서 선정된 논문들 각각에 대한 질적 평가를 하는 것이며, 통계적으로 검정할 가설은 아니다. 유사성 여부를 점검하기 위해서 PICO (Population, Intervention, Comparison, Outcome) 기법을 적용한다 [26]. 대상 연구들의 임상적 특성, 개입치료법, 비교치료법, 측정 결과의 4가지 항목에서 유사함을 확인하며, 이 과정에서 유사성을 확보하지 못한다면 나머지 두 가정에도 부정적인 영향을 미칠 뿐만 아니라 [9], 이질성 (heterogeneity) 오류도 검토해야 한다 [10,23].

(2) 이행성 (Transitivity)

앞서 유사성이 연구방법론적 비교 가능성이라면, 이행성은 논리추론적 타당성 여부이다. 간단히 설명하자면 동일 질병에 처방하는 3가지 약물 A, B, C에서 직접 비교연구 결과 A가 B보다 효과가 크고, B가 C보다 효과가 크다면, 실제 연구를 하지 않았지만 간접적으로 A가 C보다 효과가 크다는 결과를 예상할 수 있다. 이러한 이행성은 NMA 내 모든 경우에서도 지켜져야 한다 [4,5,28].

논리추론에 따라 직접비교 결과와 간접비교 결과 간을 상호 비교해 본다면 이행성 가정 여부를 객관적으로 측정할 수 있다. 이렇게 직접과 간접비교 결과를 통계학적으로 확인하는 것을 일관성이라 한다 [4,5,10]. 따라서 비일관성 (inconsistency)으로 나올 경우, 비이행성을 같이 의심해야 한다 [29]. 만약 비이행성이 의심된다면, 치료효과에 영향을 미치는 효과수정 변수 (effect modifiers)가 작용하는가에 대하여 우선 검토해야 한다 [9,27,30].

(3) 일관성 (Consistency)

이행성의 객관적 측정인 일관성은 직접 비교와 간접 비교를 통한 효과크기가 일치함을 의미한다. 따라서 이행성의 통계학적 확인이 곧 일관성이며 [5,7,24,31], 일부에서는 Coherence 라고도 한다 [8,21,32]. 이런 이유로 이행성과 일관성을 유사한 가정으로 볼 수 있다 [9,33,34]. 그러나 저자들은 이행성과 일관성은 논리추론 측면과 통계분석 측면에서 제시한 가정임을 강조하고자 이를 구분하였다.

기존에 NMA를 적용하여 발표된 논문들에 있어 일관성 가정을 확인하는 방법들을 정리해 보았을 때 [2], 통계적 모델의 적합도 (fitness)를 평가하는 것 [34,35]과 그래프의 논리적 구조를 평가하는 것[29]으로 대분할 수 있다. STATA 통계 프로그램에서 제시하는 모델 [Y]은 알아보려는 치료효과의 크기 [D]와 함께 메타분석이라면 고려해야 할 이질성 (heterogeneity) 수준 [H]과 네트워크 내 다중 비교를 위한 비일관성 수준 [C]을 동시에 고려하는 것이다 [16]. 연구내 변량 [E] 까지 고려한 식 Y = D + H + C + E 를 비일관성 모델 (inconsistency model)이라 한다. 만약 비일관성 수준이 없다고 전제할 경우 [C=0] 일관성 모델 (consistency model)이라고 따로 구분한다.

Volume: 39, Article ID: e2017047
https://doi.org/10.4178/epih.e2017047

통계학적으로 직접-간접 비교간 효과크기 차이의 신뢰구간을 기준으로 일관성 여부를 평가하는데 [10,32], NMA 연구의 약 1/8에서 비일관성을 보인다 [23]. 비일관성이 생긴 이유를 알아내는 것은 매우 중요하다 [1,10,23]. Ioannidis [36]는 비일관성을 보이는 이유들을 4가지 - 우연, 직접비교의 오류, 간접비교의 오류, 실제 다양성-로 대분하여 제시하였으며, Higgins 등 [35]은 직접비교와 간접비교의 차이를 의미하는 loop inconsistency 와 치료법간 비교하는 조합에 따른 차이인 design inconsistency 로 양분하였다.

STATA는 두 가지 수준에서 비일관성 여부를 검정한다 [12,16]. 첫째는 치료법간 비교종류별 비일관성 수준을 모두 산출한 뒤 이 값들을 Wald test 통하여 전체 연구에 대한 선형성을 검정하는 것이다. 이를 통해 전체적으로 비일관성 여부를 평가하는 전체점검 (global approach)을 한다. 둘째는 각 치료법을 따로 떼내어 (node-splitting) 직접 비교와 간접 비교의 결과값을 통계적으로 검정하는 국소점검 (local approach)을 한다.

연구자로서는 일관성 가정에 있어 관심을 가지고 탐색해야 할 것은 전체점검에서 비일관성을 야기하는 효과수정변수의 존재 여부이다 [12,37,38]. 이를

확인하기 위하여 민감도 분석을 적용하며 [3,30], 만약 효과수정 변수가 작용한다고 판단이 되면 해당 변수를 보정하기 위하여 메타회귀분석 (meta-regression)을 제안한다 [3,5,38].

다. 네트워크 지형도 (Network geometry)

NMA 분석을 위해 선정된 논문들 간의 상호 관계를 도식화한 것이다 [39]. 분석전략의 수립과 결과해석에 중요한 정보들을 제공하기에 [5,8,39], NMA 분석 결과를 발표할 때 지형도 작성을 적극 권하고 있다 [1,18]. 새로운 연구 결과나 비교하려는 치료법이 추가되면 전체적으로 지형도가 변화될 수 있다는 점은 NMA의 특징으로 볼 수 있다 [39].

STATA 적용의 실제

가. 분석준비 단계: 정보 추출 및 NMA 지원 프로그램 장착

Volume: 39, Article ID: e2017047
https://doi.org/10.4178/epih.e2017047

NMA 수행의 실제 사례를 보여주기 위해 설정한 연구 가설은 인공고관절 전치환술 (Total hip joint replacement) 시행에서 Tranexamic acid 약물 사용법에 따라 수혈률 (transfusion rate)이 차이나는 가를 알아보는 것이다. 검색과정을 거쳐 25편의 논문이 선정되었고, 추출된 결과는 별첨 1과 같다. 약물 사용법은 위약군 (placebo) A, 정맥 1회 주사법 (IV single use) B, 정맥 2회 주사법 (IV double use) C, 국소 도포법 (topical use) D, 정맥과 국소도포 복합 처치군 E의 총 5 치료법으로 분류하였다.

별첨 1의 표에서 보듯이 한 논문에서 치료법에 맞추어 대상수를 나누어 입력하는 세로 형태 (long form)로 코딩하기를 권한다. 이 형태가 명령문을 이해하고 수정하기 수월하기 때문이다.

STATA에서는 NMA 분석을 하려면 network package를 설치해야 한다 [16]. 설치 후 분석할 자료에 입력한 변수들의 의미를 지정하기 위하여 <**network setup d n, studyvar(study) trtvar(trt) ref(A)**> 란 명령어를 우선 입력한다. 명령어 중 <network setup>는 network package를 사용하도록 만드는 것이다. 차례대로 event 수 <d>와 전체 표본수 <n>를 기입한다. 콤마 뒤로는 관련 옵션들이 위치한다.

Volume: 39, Article ID: e2017047
https://doi.org/10.4178/epih.e2017047
<studyvar>는 연구명 변수를, <trtvar>은 치료법 변수를, <ref>는 치료법 중 기준치료 (reference treatment)의 변수를 각각 지정하는 것이다. 별첨 1의 정리된 자료에 해당 명령어를 입력한 결과는 Fig 2. 와 같다.

Fig 2.을 보면 기준 치료로 A (Placebo)가 설정되어 있다. 전체 25개의 연구중 Xie 2016과 Yamasaki 2004는 셀에 0이 포함되어 있기 때문에 STATA는 이를 default로 0.5 설정하며 중재군과 대조군이 각각 0.5를 가지기에 각 중재별 표본 수는 1씩 증가하였다. 또한 North 2016과 Xie 2016 처럼 기준치료 A에 관한 정보가 없을 경우 STATA는 default로 매우 작은 양의 데이터를 기준중재 변수 (reference arm)로 생성시키는데 이를 augmented 방법이라고 한다. 전체 효과크기에는 영향을 미치지 않고 수식에 오류를 줄일 수 있으며 개별연구를 모두 활용할 수 있는 장점을 갖는다.

나. (1단계) 네트워크 지형도 작성

치료법들 간의 비교 관계를 탐색하기 위하여 네크워크 지형도를 그리는 명령어는 **<network map>** 이며, 그 결과는 Fig 1. 과 같다. 각 치료법을 표시하는 5가지 노드 (node)의 크기는 해당 노드가 포함된 연구의 수를 의미하며, 노드 간을 연결하는 선의 굵기는 관련한 데이터의 양을 나타낸다. 또한 5개 노드 모두 닫힌 형태로 MTC 분석이 가능함을 확인할 수 있다. 치료법들 간의 기여도 (contribute)를 표 형태로 알아보려면 **<netweight>** 명령어를 사용한다.

다. (2단계) 비일관성 여부 확인

NMA 수행에서 요구되는 3가지 가정 중 일관성 충족 여부를 통계적으로 검정하는 단계이다. 비일관성의 전체 (overall) 점검을 위하여, STATA가 제공하는 비일관성 모델을 사용하려면 <**network meta inconsistency>** 명령어를 입력하며 그 결과는 Fig 3. 이다. Fig. 3의 가장 마지막 부분에서 제시한 p-value 는 전체점검에서 비일관성 여부 (testing for inconsistency) 를 검정한 결과이다. 귀무가설을 기각하지 못하면서, 치료별 전체 수준에서 일관성 가정을 수용할 수 있겠다.

Volume: 39, Article ID: e2017047
https://doi.org/10.4178/epih.e2017047

다음으로 Loop inconsistency를 알아보는 국소 (local) 점검을 위해 **<network sidesplit all>** 명령어가 있다. Table 1은 결과를 담은 표이다. 치료법별로 차이의 크기와 함께 통계적 검정 결과도 같이 제시하고 있다. 모든 치료법 간에 통계적 유의성을 보여주지 않고 있다. 이처럼 전체점검과 국소점검 모두에서 비일관성이 없다고 나왔기 때문에 본 연구는 일관성 가정을 수용한다.

라. (3단계) 치료법별 효과크기 그림 (plot) 및 산출 표 (league table)

효과크기를 그림과 표로 제시하기 위해선 **<network meta consistency>** 명령어를 적용해서 결과값을 메모리에 상주시켜 놓아야 한다. NMA에서 개별 연구별, 치료법별 효과 크기를 그림으로 나타내려면 네트워크 숲그림 (network forest plot, NFP) 과 간격그림 (interval plot)의 두 종류가 있다. STATA에서 NFP 작성을 위해서 **<network forest, msize(*0.15) diamond eform xlabel(0.1 1 10 100) colors(black blue red) list>** 라는 명령어를 입력한다. <network forest>는 forest plot을 그리라는 주 명령어이며, 콤마 뒤로는 옵션들을 지정한다. <diamond>는 통합효과 크기 (summary effect size)를 diamond 형태로 나타내며, <eform>은 forest plot을 해석하기 수월하게 지수 변환하여 나타낸다. 나머지는

Volume: 39, Article ID: e2017047
https://doi.org/10.4178/epih.e2017047
모두 그래프를 보기 쉽게 하기 위한 것으로서 <msize(*0.15)>는 개별연구들의 효과크기를 해당 값에서 0.15배 정도 크기로 하며, <xlabel(0.1 1 10 100)>은 x축의 표시할 단위를 설정하며, <colors(black blue red)>는 비교할 치료법 내 개별 논문별 효과, 비교할 치료법 내 통합효과 (pooled within design), 전체 수준의 통합 크기(pooled overall)의 색깔을 black, blue, 그리고 red로 설정하는 것이다 (Fig 4).

이렇게 작성한 NFP에서 다양한 정보를 알아 볼 수 있다. 첫째, 개별 연구와 각 치료법에 효과크기를 알 수 있다. 비교할 치료법 내 통합효과 크기 (blue color)는 비일관성 검정 모델을, 전체 수준의 통합 크기(red color)는 일관성 검정모델을 적용한 결과 값을 제시한 것이다. 둘째, 그림의 좌측 하단에 제시한 p-값은 앞서 비일관성 검정의 전체점검 결과와 일치하며, 이를 근거로 일관성을 수용할 수 있음을 확인할 수 있다. 셋째, 육안으로 비교치료법 내 개별 연구들 간의 이질성 여부를 확인할 수 있을 뿐만 아니라, 비교할 치료법 내 통합효과 크기 (blue color)와 전체 수준의 통합 크기(red color) 간의 유사성을 근거로 일관성 모델을 지지한다고 판단할 수 있다.

Volume: 39, Article ID: e2017047
https://doi.org/10.4178/epih.e2017047

이처럼 NFP 가 유용한 정보들을 주는 것은 사실이나, 선정한 분석 논문이 많고 치료법 종류가 많아지면 가독성이 떨어지게 된다. 이럴 경우 interval plot을 작성하길 권한다. 이를 위한 명령어는 <**intervalplot, eform null(1) labels(Placebo IV_single IV_double Topical Combination) separate margin(10 8 5 10) textsize(2) xlabel(0.01 0.1 1 10)>** 이다. <intervalplot>은 주 명령어이며, <eform>은 원자료가 로그수치이므로 해석을 위해서 지수변환을 실시하고, <null(1)>은 교차비 (odds ratio) 같은 비에서 통계적 유의차를 나타내는 값인 1을 입력하며, <label>은 치료법 표시를 정하는 것이다. <separate>와 <margin>은 그림을 보기 쉽게 하려고 범위를 지정하는 것으로, 논문의 개수와 비교하는 치료법 종류에 따라서 편차가 너무 심하여 사용자가 적절히 조절한다. Fig 5. 는 위에서 입력한 명령어로 얻은 interval plot이다. 각 치료법별로 효과크기의 비교가 직관적이며 해석하기에 매우 용이하다는 장점을 가진다. 위의 Intervalplot 명령어 실시한 후 결과 창에 표시되는 중재별 효과크기 비교 결과를 이용하여 별첨 2 같은 network league table (NLT)을 만들 수 있다.

마. (4단계) 비교우위 치료법 선정

Volume: 39, Article ID: e2017047
https://doi.org/10.4178/epih.e2017047

이상의 분석 단계를 거쳐서 치료법별 비교로 효과 차이를 확인하였다면, 비교한 치료법들 중 우열을 가려서 선정 순위를 정할 필요가 있다 [12]. 다시 말해서 치료법 결정을 위해 어느 치료법의 효과가 가장 우수한가를 평가할 필요가 있다.

STATA 에서는 치료별 순위를 산출하도록 두 가지 명령어 - network rank와 SUCRA –를 지원한다. 두 명령어 모두 결과에 있어서는 별 차이를 보이지 않는다. 사용하기 더 용이한 network rank 수행을 위한 명령어 입력은 **<network rank min, line cumulative xlabel(1/4) seed(10000) reps(10000) meanrank>**이다. <network rank>는 주 명령어 이며, <min/max> 중에서 효과크기가 작을수록 우수한 것인지 클수록 우수한 것인지 방향을 설정한다. 적용 사례는 기준치료 (위약군 A)에 대비하여 작을수록 우수한 것이니 min을 설정하였다.

Fig 6에서 볼 수 있듯이 E(combination)가 최선 (best) 치료일 확률이 98.1%이며, 두 번째까지 최선일 확률이 약 99.2%이다. 누적순위확률 곡선하 면적(the surface under the cumulative ranking, SUCRA)에서 E 치료법이 거의 100%에 이르러 가장 좋은 중재임을 재확인 된다 [40]. 보다 정밀한 누적순위확률 계산을 위해서

Volume: 39, Article ID: e2017047
https://doi.org/10.4178/epih.e2017047

SUCRA 명령어를 적용한다. SUCRA 결과상 E 치료법 다음으로 C(IV double), D(Topical), B(IV single), 그리고 A(Placebo)의 순위를 보이고 있다. 이 결과를 임상적으로 해석한다면 인공관절 전치환술 후 수혈받을 확률을 가장 낮추기 위해서는 Tranexamic acid의 combination 사용을 권한다.

바. (5단계) 출판오류 점검

NMA에서 출판오류 (Publication bias)를 확인하기 위하여 network funnel plot 을 작성한다. 그러나 STATA network package에서 바로 그릴 수가 없기 때문에, 별첨 2처럼 분석 자료를 변형해야 한다. 이를 위하여 network forest 명령의 list 옵션을 주었을 때 나온 결과 데이터를 확보한 다음, 논문별로 직접 비교하려는 치료법별 (t1, t2)로 효과크기 (diff)와 표준오차 (se)을 정리한다.

별첨 3의 데이터를 STATA 새 창에 올린 다음, **<netfunnel diff se t1 t2, random bycomparison>** 명령어를 입력하면 network funnel plot를 얻어낼 수 있다. <netfunnel>은 주 명령어 이며, <diff>는 치료법간 로그변환된 효과크기이며 <se>는 표준오차를 지정한다. 옵션의 <random>은 변량효과모형 (random effect

Volume: 39, Article ID: e2017047
https://doi.org/10.4178/epih.e2017047

model)을 사용하며 <bycomparison>은 비교하는 치료종류별로 다른 색으로 구별하여 제시하라는 것이다. 만약 위약군 A에 대하여 선별적으로 funnel plot을 원한다면 <**netfunnel diff se t1 t2 if t1=="A", random bycomparison>** 라는 명령어를 입력한다. 얻어진 그림에 있어 출판오류의 해석은 대칭성을 기준으로 시각적으로 판단한다. 필요에 따라서는 민감도 분석을 고려하기를 권고한다.

Volume: 39, Article ID: e2017047
https://doi.org/10.4178/epih.e2017047

토론 및 제언

지금까지 빈도주의 틀에 기반한 NMA 적용 과정을 설명하였다. NMA를 적용할 연구자에게 NMA 연구 수행에 있어서 고려할 점과 이에 대한 제안점을 나열하면 다음과 같다.

첫째, 동일한 치료법들의 직접-간접 비교를 하는 것으로 RCT 연구설계를 적용한 연구결과에 국한된다 [5]. 현재의 개발 수준으로는 코호트 연구나 환자-대조군연구 등의 관찰연구결과를 NMA를 적용하지 않는 것을 권한다. 한편, RCT 연구 수행 주체, 연구비 지원자, 국가별 시판허가된 약물의 종류, 연구윤리 운영제도 등이 RCT 연구내용 및 결과에 영향을 준다는 점도 감안할 필요가 있다 [41].

둘째, 충족해야 할 가정 3가지를 치밀하게 확인해야 한다 [25,42]. 그 중, 유사성과 이행성에 있어서 임상가 입장에서 실질적으로 판단을 해야 한다 [42]. 통계적 검정으로 비일관성이 확인된다면, 연구자는 네트워크 지형도를 활용하여

분석대상 연구들의 관계들을 면밀히 조사해서 효과수정변수를 찾아내어야 한다 [37]. 그런 다음 NMA 메타회귀분석 등을 적용하여 해당 변수의 보정 전후의 결과를 비교 평가해서 임상적으로 타당한 추론을 도출하는 노력이 필요하다 [5,31].

셋째, 소규모 시험들에 의한 오류 개입 여부를 고려해야 한다 [8]. 대상자수가 작은 연구는 출판오류를 야기시킬 뿐만 아니라 [43], 상대적으로 큰 치료효과를 산출할 수 있기 때문이다 [44]. 랜덤효과모형을 적용하고 [7], 민감도 분석을 통해 오류 여부를 평가하기를 권한다 [3,30].

넷째, 빈도주의 틀에 따른 NMA 분석의 한계를 감안해서 베이지언 틀을 적용하는 것을 고려해 볼 필요가 있다 [6,7]. 불확실성 (uncertainty) 속에서 보건의료의 의사결정에 적용하고 그 결과를 예측하는 것이 NMA 연구의 최종 목적이라면, 베이지언 분석이 보다 적합하기 때문이다 [11-13]. 현재 WinBUGS (https://www.mrc-bsu.cam.ac.uk/software/bugs/) 등의 베이지언 분석을 지원하는 여러 통계 프로그램이 개발되어 있다 [12].

과학적 설득력 수준에서 메타분석을 적용한 체계적 고찰을 통해 종합한 근거는 과학적 설득력 수준에서 가장 상위에 위치한다 [45]. 더구나 동일한 치료 목적을 가진 여러 치료약들 간의 직-간접 비교를 통해 처치효과를 종합하고 우선순위를 제시하는 NMA로 얻어낸 결과는 보건의료에서 근거중심의 의사결정에 큰 의미를 갖는다 [3,5,46]. 따라서 우리나라 보건의료 수준의 질적 향상을 위해서 NMA는 활성화될 필요가 있다. 본 원고를 통해 국내 연구자들이 NMA 수행을 촉진하는데 일조하기를 기대한다.

감사의 글

본 원고는 메타분석연구회(신인수 회장) 연구활동의 결과물임을 밝힙니다.

Volume: 39, Article ID: e2017047
https://doi.org/10.4178/epih.e2017047

Volume: 39, Article ID: e2017047
https://doi.org/10.4178/epih.e2017047

References

1. Mills EJ, Ioannidis JP, Thorlund K, Schünemann HJ, Puhan MA, Guyatt GH. How to use an article reporting a multiple treatment comparison meta-analysis. JAMA. 2012;308(12):1246-53.
2. Donegan S, Williamson P, D'Alessandro U, Tudur Smith C. Assessing key assumptions of network meta-analysis: a review of methods. Res Synth Methods. 2013;4(4):291-323.
3. Biondi-Zoccai G, Abbate A, Benedetto U, Palmerini T, D'Ascenzo F, Frati G. Network meta-analysis for evidence synthesis: what is it and why is it posed to dominate cardiovascular decision making? Int J Cardiol. 2015;182:309-14.
4. Caldwell DM. An overview of conducting systematic reviews with network meta-analysis. Syst Rev. 2014;3:109.
5. Cipriani A, Higgins JP, Geddes JR, Salanti G. Conceptual and technical challenges in network meta-analysis. Ann Intern Med. 2013;159(2):130-7.
6. Lu G, Ades AE. Combination of direct and indirect evidence in mixed treatment comparisons. Stat Med. 2004;23(20):3105-24.
7. Jansen JP, Fleurence R, Devine B, Itzler R, Barrett A, Hawkins N, et al. Interpreting indirect treatment comparisons and network meta-analysis for health-care decision making: report of the ISPOR Task Force on Indirect Treatment Comparisons Good Research Practices: part 1. Value Health. 2011;14(4):417-28.
8. Mills EJ, Thorlund K, Ioannidis JP. Demystifying trial networks and network meta-analysis. BMJ. 2013;346:f2914.
9. Jansen JP, Naci H. Is network meta-analysis as valid as standard pairwise meta-analysis? It all depends on the distribution of effect modifiers. BMC Med. 2013;11:159.
10. Salanti G. Indirect and mixed-treatment comparison, network, or multiple-treatments meta-analysis: many names, many benefits, many concerns for the next generation evidence synthesis tool. Res Synth Methods. 2012;3(2):80-97.
11. Dakin HA, Welton NJ, Ades AE, Collins S, Orme M, Kelly S. Mixed treatment comparison of repeated measurements of a continuous endpoint: an example using topical treatments for primary open-angle glaucoma and ocular hypertension. Stat Med. 2011;30(20):2511-35.
12. Tonin FS, Rotta I, Mendes AM, Pontarolo R. Network meta-analysis: a technique to gather evidence from direct and indirect comparisons. Pharm Pract (Granada). 2017;15(1):943.

Volume: 39, Article ID: e2017047
https://doi.org/10.4178/epih.e2017047

13. Schmitz S, Adams R, Walsh CD, Barry M, FitzGerald O. A mixed treatment comparison of the efficacy of anti-TNF agents in rheumatoid arthritis for methotrexate non-responders demonstrates differences between treatments: a Bayesian approach. Ann Rheum Dis. 2012;71(2):225-30.
14. Bucher HC, Guyatt GH, Griffith LE, Walter SD. The results of direct and indirect treatment comparisons in meta-analysis of randomized controlled trials. J Clin Epidemiol. 1997;50(6):683-91.
15. Jones B, Roger J, Lane PW, Lawton A, Fletcher C, Cappelleri JC, et al. Statistical approaches for conducting network meta-analysis in drug development. Pharm Stat. 2011;10(6):523-31.
16. White IR. Network meta-analysis. Stata J. 2015;15(4):951-85.
17. Hoaglin DC, Hawkins N, Jansen JP, Scott DA, Itzler R, Cappelleri JC, et al. Conducting indirect-treatment-comparison and network-meta-analysis studies: report of the ISPOR Task Force on Indirect Treatment Comparisons Good Research Practices: part 2. Value Health. 2011;14(4):429-37.
18. Hutton B, Salanti G, Caldwell DM, Chaimani A, Schmid CH, Cameron C, et al. The PRISMA extension statement for reporting of systematic reviews incorporating network meta-analyses of health care interventions: checklist and explanations. Ann Intern Med. 2015;162(11):777-84.
19. Li T, Puhan MA, Vedula SS, Singh S, Dickersin K; Ad Hoc Network Meta-analysis Methods Meeting Working Group. Network meta-analysis-highly attractive but more methodological research is needed. BMC Med. 2011;9:79.
20. Hasselblad V. Meta-analysis of multitreatment studies. Med Decis Making. 1998;18(1):37-43.
21. Lumley T. Network meta-analysis for indirect treatment comparisons. Stat Med. 2002;21(16):2313-24.
22. Shim SR, Shin IS, Bae JM. Intervention meta-analysis using STATA software. J Health Info Stat. 2016;41(1):123-34.
23. Veroniki AA, Vasiliadis HS, Higgins JP, Salanti G. Evaluation of inconsistency in networks of interventions. Int J Epidemiol. 2013;42(1):332-45.
24. Bhatnagar N, Lakshmi PV, Jeyashree K. Multiple treatment and indirect treatment comparisons: An overview of network meta-analysis. Perspect Clin Res. 2014;5(4):154-8.
25. Mills EJ, Bansback N, Ghement I, Thorlund K, Kelly S, Puhan MA, et al. Multiple treatment comparison meta-analyses: a step forward into complexity. Clin Epidemiol. 2011;3:193-202.

26. Reken S, Sturtz S, Kiefer C, Böhler YB, Wieseler B. Assumptions of Mixed Treatment Comparisons in Health Technology Assessments - Challenges and Possible Steps for Practical Application. PLoS One. 2016;11(8):e0160712.
27. Salanti G, Marinho V, Higgins JP. A case study of multiple-treatments meta-analysis demonstrates that covariates should be considered. J Clin Epidemiol. 2009;62(8):857-64.
28. Baker SG, Kramer BS. The transitive fallacy for randomized trials: if A bests B and B bests C in separate trials, is A better than C? BMC Med Res Methodol. 2002;2:13.
29. Krahn U, Binder H, König J. A graphical tool for locating inconsistency in network meta-analyses. BMC Med Res Methodol. 2013;13:35.
30. Dias S, Welton NJ, Sutton AJ, Caldwell DM, Lu G, Ades AE. Evidence synthesis for decision making 4: inconsistency in networks of evidence based on randomized controlled trials. Med Decis Making. 2013;33(5):641-56.
31. Lu G, Ades AE. Assessing evidence inconsistency in mixed treatment comparisons. J Am Stat Assoc. 2006;101(474):447-59.
32. Cipriani A, Furukawa TA, Salanti G, Geddes JR, Higgins JP, Churchill R, et al. Comparative efficacy and acceptability of 12 new-generation antidepressants: a multiple-treatments meta-analysis. Lancet. 2009;373(9665):746-58.
33. Chaimani A, Salanti G, Leucht S, Geddes JR, Cipriani A. Common pitfalls and mistakes in the set-up, analysis and interpretation of results in network meta-analysis: what clinicians should look for in a published article. Evid Based Ment Health. 2017 Jul 24. pii: ebmental-2017-102753. doi: 10.1136/eb-2017-102753.
34. Dias S, Welton NJ, Caldwell DM, Ades AE. Checking consistency in mixed treatment comparison meta-analysis. Stat Med. 2010;29(7-8):932-44.
35. Higgins JP, Jackson D, Barrett JK, Lu G, Ades AE, White IR. Consistency and inconsistency in network meta-analysis: concepts and models for multi-arm studies. Res Synth Methods. 2012;3(2):98-110.
36. Ioannidis JP. Integration of evidence from multiple meta-analyses: a primer on umbrella reviews, treatment networks and multiple treatments meta-analyses. CMAJ. 2009;181(8):488-93.
37. Saramago P, Sutton AJ, Cooper NJ, Manca A. Mixed treatment comparisons using aggregate and individual participant level data. Stat Med. 2012;31(28):3516-36.
38. White IR, Barrett JK, Jackson D, Higgins JP. Consistency and inconsistency in network meta-analysis: model estimation using multivariate meta-regression. Res Synth Methods. 2012 Jun;3(2):111-25.

39. Salanti G, Kavvoura FK, Ioannidis JP. Exploring the geometry of treatment networks. Ann Intern Med. 2008;148(7):544-53.
40. Salanti G, Ades AE, Ioannidis JP. Graphical methods and numerical summaries for presenting results from multiple-treatment meta-analysis: an overview and tutorial. J Clin Epidemiol. 2011;64(2):163-71.
41. Ioannidis JP. Indirect comparisons: the mesh and mess of clinical trials. Lancet. 2006;368(9546):1470-2.
42. Kyrgiou M, Salanti G, Pavlidis N, Paraskevaidis E, Ioannidis JP. Survival benefits with diverse chemotherapy regimens for ovarian cancer: meta-analysis of multiple treatments. J Natl Cancer Inst. 2006;98(22):1655-63.
43. Ioannidis JP, Cappelleri JC, Lau J. Issues in comparisons between meta-analyses and large trials. JAMA. 1998;279(14):1089-93.
44. Pereira TV, Horwitz RI, Ioannidis JP. Empirical evaluation of very large treatment effects of medical interventions. JAMA. 2012;308(16):1676-84.
45. Bae JM. Meta-epidemiology. Epidemiol Health. 2014;36:e2014019.
46. Bae JM. Suggestions for the promotion of evidence-based public health in South Korea. Epidemiol Health. 2017;39:e201703.

Volume: 39, Article ID: e2017047
https://doi.org/10.4178/epih.e2017047

Table 1. Inconsistency test between direct and indirect treatment comparison in mixed treatment comparison

Side	Direct		Indirect		Difference		
	Coef.	Std. Err.	Coef.	Std. Err.	Coef.	Std. Err.	P>z
A B	-1.083	0.174	-0.877	0.620	-0.206	0.636	0.746
A C	-1.388	0.247	-1.869	0.493	0.481	0.542	0.375
A D	-1.378	0.265	-0.738	0.413	-0.640	0.479	0.182
A E	-3.425	0.940	-3.221	1.005	-0.204	0.937	0.828
B C	-0.894	0.655	-0.312	0.297	-0.581	0.715	0.416
B D	0.099	0.462	-0.241	0.329	0.340	0.567	0.548
B E	-2.152	0.881	-2.615	1.087	0.463	0.896	0.605
C D	0.490	0.492	0.177	0.350	0.313	0.604	0.605
D E	-2.550	1.254	-1.956	0.958	-0.595	1.314	0.651
