# Supplementary material for: Network meta-analysis: application and practice using Stata
Source: Epidemiol Health. 2017 Oct 27;39:e2017047. doi: 10.4178/epih.e2017047 (PMC5733388; doi:10.4178/epih.e2017047)
Supplement: Supplementary file 2 [file epih-39-e2017047-app1.pdf]

Appendix 1. The extracted information from the selected articles for conducting a network meta-analysis using STATA*

study	d	n	trt
Alshryda 2013	10	80	C
Alshryda 2013	26	81	A
Barrachina 2016	8	35	E
Barrachina 2016	4	36	B
Barrachina 2016	14	37	A
Benoni 2000	9	20	B
Benoni 2000	15	19	A
Benoni 2001	4	18	E
Benoni 2001	8	20	A
Claeys 2007	1	20	E
Claeys 2007	6	20	A
Ekbäck 2000	1	20	B
Ekbäck 2000	1	20	A
Fraval 2017	1	50	B
Fraval 2017	6	51	A
Garneti 2004	16	25	E

Garneti 2004	14	25	A
Husted 2003	2	20	B
Husted 2003	7	20	A
Hsu 2015	2	30	B
Hsu 2015	9	30	A
Johansson 2005	8	47	E
Johansson 2005	23	53	A
Kazemi 2010	7	32	E
Kazemi 2010	15	32	A
Lee 2013	9	34	B
Lee 2013	20	34	A
Lemay 2004	6	20	B
Lemay 2004	13	19	A
Martin 2014	3	25	C
Martin 2014	5	25	A
Na 2016	2	29	E
Na 2016	5	26	A
Niskanen 2005	5	19	B
Niskanen 2005	8	20	A

North 2016	8	70	B
North 2016	12	69	C
Rajesparan 2009	3	36	E
Rajesparan 2009	10	37	A
Wang 2016	9	81	E
Wang 2016	10	38	A
Wei 2014	6	101	E
Wei 2014	6	102	C
Wei 2014	26	100	A
Xie 2016	3	70	E
Xie 2016	4	70	C
Xie 2016	0	70	D
Yi 2016	8	50	E
Yi 2016	1	50	D
Yi 2016	19	50	A
Yamasaki 2004	0	20	E
Yamasaki 2004	0	20	A
Yue 2014	3	52	C

Yue 2014	11	49	A

* d, events. n, total sample sizes. trt, treatment.; A, Placebo; B, IV(single); C, IV(double); D, Topical; E, Combination

[Alshryda 2013] Alshryda S, Mason J, Sarda P, Nargol A, Cooke N, Ahmad H, et al. Topical (Intra-Articular) Tranexamic Acid Reduces Blood Loss and Transfusion Rates Following Total Hip Replacement. J Bone Joint Surg Am 2013;95;1969-74.

[Barrachina 2016] Barrachina B, Lopez-Picado A, Remon M, Fondarella A, Iriarte I, Bastida R, et al. Tranexamic Acid Compared with Placebo for Reducing Total Blood Loss in Hip Replacement Surgery: A Randomized Clinical Trial. AnesthAnalg 2016;122;986-95.

[Benoni 2000] Benoni G, Lethagen S, Nilsson P, Fredin H. Tranexamic acid, given at the end of the operation, does not reduce postoperative blood loss in hip arthroplasty. Acta OrthopScand 2000;71;250-4.

[Benoni 2001] Benoni G, Fredin H, Knebel R, Nilsson P. Blood conservation with tranexamic acid in total hip arthroplasty: a randomized, double-blind study in 40 primary operations. ActaOrthopScand 2001;72;442-8.

[Claeys 2016] Claeys MA, Vermeersch N, Haentjens P. Reduction of Blood Loss with Tranexamic Acid in Primary Total Hip Replacement Surgery. ActaChirurgicaBelgica2016;107:397-401.

[Ekback 2000] Ekback G, Axelsson K, Ryttberg L, Edlund B, Kjellberg J, Weckstrom J, et al. Tranexamic acid reduces blood loss in total hip replacement surgery. AnesthAnalg2000;91:1124-30.

[Fraval 2017] Fraval A, Effeney P, Fiddelaers L, Smith B, Towell B, Tran P. OBTAIN A: Outcome Benefits of Tranexamic Acid in Hip Arthroplasty. A Randomized Double-Blinded Controlled Trial. J Arthroplasty 2017;32:1516-19.

[Garneti 2004] Garneti N, Field J. Bone bleeding during total hip arthroplasty after administration of tranexamic acid. J Arthroplasty 2004;19:488-92.

[Hsu 2015] Hsu CH, Lin PC, Kuo FC, Wang JW. A regime of two intravenous injections of tranexamic acid reduces blood loss in minimally invasive total hip arthroplasty: a prospective randomised double-blind study. Bone Joint J. 2015;97-B:905-10.

[Husted 2003] Husted H, Blond L, Sonne-Holm S, Holm G, Jacobsen TW, Gebuhr P. Tranexamic acid reduces blood loss and blood transfusions in primary total hip arthroplasty: a prospective randomized double-blind study in 40 patients. ActaOrthopScand2003;74:665-9.

[Johansson 2005] Johansson T, Pettersson LG, Lisander B. Tranexamic acid in total hip arthroplasty saves blood and money: a randomized, double-blind study in 100 patients. ActaOrthop2005;76:314-9.

[Kazemi 2010] Kazemi SM, Mosaffa F, Eajazi A, Kaffashi M, Besheli LD, Bigdeli MR, et al. The Effect of Tranexamic Acid on Reducing Blood Loss in Cementless Total Hip Arthroplasty Under Epidural Anesthesia. Orthopedics 2010;33:17-22.

[Lee 2013] Lee YC, Park SJ, Kim JS, Cho CH. Effect of tranexamic acid on reducing postoperative blood loss in combined hypotensive epidural anesthesia and general anesthesia for total hip replacement. J ClinAnesth2013;25:393-8.

[Lemay 2004] Lemay E, Guay J, Cote C, Roy A. Tranexamic acid reduces the need for allogenic red blood cell transfusions in patients undergoing total hip replacement. Can J Anaesth2004;51:31-7.

[Martin 2014] Martin JG, Cassatt KB, Kincaid-Cinnamon KA, Westendorf DS, Garton AS, Lemke JH. Topical administration of tranexamic acid in primary total hip and total knee arthroplasty. J Arthroplasty 2014;29:889-94.
[Na 2016] Na HS, Shin HJ, Lee YJ, Kim JH, Koo KH, Do SH. The effect of tranexamic acid on blood coagulation in total hip replacement arthroplasty: rotational thromboelastographic (ROTEM(R)) analysis. Anaesthesia2016;71:67-75.
[Niskanen 2005] Niskanen RO, Korkala OL. Tranexamic acid reduces blood loss in cemented hip arthroplasty: a randomized, double-blind study of 39 patients with osteoarthritis. ActaOrthop2005;76:829-32.
[North 2016] North WT, Mehran N, Davis JJ, Silverton CD, Weir RM, Laker MW. Topical vs Intravenous Tranexamic Acid in Primary Total Hip Arthroplasty: A Double-Blind, Randomized Controlled Trial. J Arthroplasty 2016;31:1022-6.
[Rajesparan 2009] Rajesparan K, Biant LC, Ahmad M, Field RE. The effect of an intravenous bolus of tranexamic acid on blood loss in total hip replacement. J Bone Joint Surg Br 2009;91:776-83.
[Wang 2016] Wang C, Kang P, Ma J, Yue C, Xie J, Pei F. Single-dose tranexamic acid for reducing bleeding and transfusions in total hip arthroplasty: A double-blind, randomized controlled trial of different doses. Thromb Res 2016;141:119-23.
[Wei 2014] Wei W, Wei B. Comparison of topical and intravenous tranexamic acid on blood loss and transfusion rates in total hip arthroplasty. J Arthroplasty 2014;29:2113-6.
[Xie 2016] Xie J, Ma J, Yue C, Kang P, Pei F. Combined use of intravenous and topical tranexamic acid following cementless total hip arthroplasty: a randomised clinical trial. Hip Int2016;26:36-42.
[Yamasaki 2004] Yamasaki S, Masuhara K, Fuji T. Tranexamic acid reduces blood loss after cementless total hip arthroplasty-prospective randomized study in 40 cases. IntOrthop2004;28:69-73.
[Yi 2016] Yi Z, Bin S, Jing Y, Zongke Z, Pengde K, Fuxing P. Tranexamic Acid Administration in Primary Total Hip Arthroplasty: A Randomized Controlled Trial of Intravenous Combined with Topical Versus Single-Dose Intravenous Administration. J Bone Joint Surg Am 2016;98:983-91.
[Yue 2014] Yue C, Kang P, Yang P, Xie J, Pei F. Topical application of tranexamic acid in primary total hip arthroplasty: a randomized double-blind controlled trial. J Arthroplasty 2014;29:2452-6.
