# Supplementary material for: Network meta-analysis: application and practice using Stata
Source: Epidemiol Health. 2017 Oct 27;39:e2017047. doi: 10.4178/epih.e2017047 (PMC5733388; doi:10.4178/epih.e2017047)
Supplement: Supplementary file 3 [file epih-39-e2017047-app2.pdf]

Appendix 2. A network league table based on the network meta-analysis from data in appendix 1

Placebo	CIL	CIU										
0.34	0.25	0.48	IV_single	CIL	CIU							
0.23	0.15	0.35	0.66	0.39	1.13	IV_double	CIL	CIU				
0.30	0.19	0.47	0.88	0.52	1.49	1.33	0.76	2.32	Topical	CIL	CIU	
0.04	0.01	0.19	0.10	0.02	0.55	0.16	0.03	0.87	0.12	0.02	0.64	Combination

CIL & CIU, 95% confidence interval lower and upper limitation.
