# Supplementary material for: Network meta-analysis: application and practice using Stata
Source: Epidemiol Health. 2017 Oct 27;39:e2017047. doi: 10.4178/epih.e2017047 (PMC5733388; doi:10.4178/epih.e2017047)
Supplement: Supplementary file 4 [file epih-39-e2017047-app3.pdf]

Appendix 3. The raw data for drawing a network funnel plot from data in appendix 1.

t1	t2	design	diff	se
A	B	A B	-0.847	0.728
A	B	A B	-2.097	1.136
A	B	A B	0.334	0.58
A	B	A B	-1.318	0.477
A	B	A B	-1.148	0.555
A	B	A B	-1.168	0.886
A	B	A B	-1.405	0.708
A	B	A B	-1.05	0.511
A	B	A B	0	2.024
A	B	A B C	-0.72	0.526
A	B	A B D	-1.716	0.479
A	B	A B E	-1.169	0.483
A	C	A B C	-1.583	0.629
A	C	A C	-1.522	0.72
A	C	A C	0	1.451
A	C	A C	-1.877	1.1
A	C	A C	-1.792	0.833
A	C	A C	-1.578	0.881
A	C	A C	-1.378	0.522
A	C	A C	-1.62	0.694
A	C	A C	-0.624	0.693
A	D	A B D	-1.727	0.479
A	D	A D	-1.197	0.413
A	D	A D	-0.606	0.793
A	D	A D	-1.554	0.686
A	E	A B E	-3.402	1.051
B	C	A B C	-0.863	0.666
B	D	A B D	-0.01	0.595
B	D	B D E	0.266	0.733
B	E	A B E	-2.234	1.081
B	E	B D E	-1.989	1.521
C	D	C D	0.49	0.492
D	E	B D E	-2.256	1.5
